# Mastocytosis in the skin in children and adults

**DOI:** 10.1186/2045-7022-5-S1-O11

**Published:** 2015-03-11

**Authors:** Magdalena Lange

**Affiliations:** 1 Medical University of Gdañsk Department of Dermatology Venereology and Allergology, Gdañsk, Denmark

## Background

Mastocytosis is a rare disease of bone marrow-derived hematopoietic progenitor cells, which is characterized by abnormal growth and excessive accumulation of mast cells in various tissues. Cutaneous Mastocytosis is a skin limited disease, whereas Systemic Mastocytosis usually involves bone marrow, spleen, liver, lymph nodes and gastrointestinal tract and may present with or without skin involvement. The skin is one of the most commonly affected organ both in children and adults. Mastocytosis in the skin (MIS) is defined by a typical exanthema and monomorphic mast cell infiltrate. MIS is an initial diagnosis which requires further diagnostic steps leading to the final diagnosis of Cutaneous Mastocytosis or Systemic Mastocytosis.

## Methods

The study group included 140 adult patients and 160 children presenting MIS. All patients were clinically diagnosed with Systemic Mastocytosis strictly according WHO criteria.

## Results

Cutaneous Mastocytosis was divided into maculopapular type, diffuse type and mastocytoma. Cutaneous Mastocytosis was the predominat form of the disease in children. By contrast, in most adult patients Systemic Mastocytosis was recognized. A substantial number of mastocytosis patients suffered from mediator-related symptoms including pruritus, flushing, hypotension, headache, nausea, abdominal pain, diarrhea and anaphylactic shock among others. The present study is focused on the presentation of the clinical diversity of Mastocytosis in the skin, demonstrating diagnostic algorithms and comparison of disease features in children and adults diagnosed in the Mastocytosis Centre, Medical University of Gdañsk.

## Conclusion

Due to a rare incidence of Systemic Mastocytosis in children, the diagnostic approach in children and adults presenting MIS should differ.

